# The Mitochondrial Permeability Transition Pore Regulator Cyclophilin D Exhibits Tissue-Specific Control of Metabolic Homeostasis

**DOI:** 10.1371/journal.pone.0167910

**Published:** 2016-12-22

**Authors:** Rhianna C. Laker, Evan P. Taddeo, Yasir N. Akhtar, Mei Zhang, Kyle L. Hoehn, Zhen Yan

**Affiliations:** 1 Department of Medicine, University of Virginia School of Medicine, Charlottesville, VA, United States of America; 2 Center for Skeletal Muscle Research at Robert M. Berne Cardiovascular Research Center, University of Virginia School of Medicine, Charlottesville, VA, United States of America; 3 Department of Pharmacology, University of Virginia School of Medicine, Charlottesville, VA, United States of America; 4 Department of Molecular Physiology & Biological Physics, University of Virginia School of Medicine, Charlottesville, VA, United States of America; Instituto Nacional de Cardiologia Ignacio Chavez, MEXICO

## Abstract

The mitochondrial permeability transition pore (mPTP) is a key regulator of mitochondrial function that has been implicated in the pathogenesis of metabolic disease. Cyclophilin D (CypD) is a critical regulator that directly binds to mPTP constituents to facilitate the pore opening. We previously found that global CypD knockout mice (KO) are protected from diet-induced glucose intolerance; however, the tissue-specific function of CypD and mPTP, particularly in the control of glucose homeostasis, has not been ascertained. To this end, we performed calcium retention capacity (CRC) assay to compare the importance of CypD in the liver versus skeletal muscle. We found that liver mitochondria are more dependent on CypD for mPTP opening than skeletal muscle mitochondria. To ascertain the tissue-specific role of CypD in metabolic homeostasis, we generated liver-specific and muscle-specific CypD knockout mice (LKO and MKO, respectively) and fed them either a chow diet or 45% high-fat diet (HFD) for 14 weeks. MKO mice displayed similar body weight gain and glucose intolerance compared with wild type littermates (WT), whereas LKO mice developed greater visceral obesity, glucose intolerance and pyruvate intolerance compared with WT mice. These findings demonstrate that loss of muscle CypD is not sufficient to alter whole body glucose metabolism, while the loss of liver CypD exacerbates obesity and whole-body metabolic dysfunction in mice fed HFD.

## Introduction

Impaired mitochondrial function due to increased damage and/or impaired maintenance is associated with a plethora of disease conditions affecting multiple organ systems. The contribution of mitochondrial dysfunction to obesity and type 2 diabetes is particularly important since these two related disease conditions are reaching epidemic levels and are primarily attributed to poor diet and sedentary lifestyle. A key regulator of mitochondrial health is the mitochondrial permeability transition pore (mPTP). The mPTP presumably spans both the outer and inner mitochondrial membranes and when open allows the passage of molecules up to 1.5 kDa in size [1]. The molecular constituents of the mPTP have long been debated. For a period of time the widely accepted structure included the adenine nucleotide transporter (ANT) and the voltage dependent anion channel (VDAC) [2–3]. However, gene inactivation studies found that neither of these channel-forming proteins were critical for the pore opening [4–6]. More recently, the F_O_F_1_ ATP synthase was reported to dimerize and form channels with behavior indistinguishable from that of the mPTP [7–8].

Opening of the mPTP can be triggered by a variety of events including calcium overload and exacerbated reactive oxygen species production [9–11]. Transient opening of the pore is important for release of ions and metabolites to maintain mitochondrial homeostasis [12–14]. However, under conditions of extreme cellular stress, irreversible opening of the mPTP signals mitochondrial degeneration and triggers removal of the mitochondria from the cell [15–17]. Therefore, normal mPTP sensitivity to stimuli for opening plays a critical role in maintaining normal metabolic function.

The matrix peptidyl-prolyl cis-trans isomerase, cyclophilin D (CypD) is a critical regulator of the mPTP and directly binds to pore constituents, including ANT [18] and F_O_F_1_ ATP synthase [7] to facilitate opening [19–22]. There have been conflicting reports in the literature regarding the metabolic phenotype of global CypD knockout (KO) mice under both basal and high-fat diet (HFD) conditions [23–27]. In our hands, whole body CypD KO mice displayed normal metabolic phenotype when fed normal chow [23]. However, after 11 weeks of 45% high-fat diet (HFD) CypD KO mice had better whole body glucose disposal compared with wild type (WT) littermates. This resistance of CypD KO mice to developing glucose intolerance following high-fat feeding was attributed to improved skeletal muscle uptake of radiolabelled glucose following an i.p. bolus injection of glucose [23]. Yet, there was a tendency for these mice to have poor glucose disposal in the liver [23]. Another study reported similar findings of improved glucose tolerance in CypD KO mice [24], with others reporting resistance to HFD-induced obesity, but with severe glucose intolerance compared to WT [25]. Two other studies have found that mice lacking CypD displayed adult onset obesity with increased adipose deposition in the absence of increased food intake fed normal diet [26], and hepatic insulin resistance at 24 weeks of age [27].

In an attempt to understand and dissect these inconsistencies, we assessed calcium loading-induced mPTP in mitochondria isolated from skeletal muscle and liver from global CypD KO mice and compared with those from wild type littermates. We also generated mice with either muscle-specific or liver-specific deletion of CypD (MKO and LKO, respectively) to determine their glucose tolerance when fed normal chow or 45% HFD. Our findings suggest that CypD deletion specifically in skeletal muscle has minimal metabolic consequences, whereas hepatic-specific deletion of the CypD gene led to exacerbated impairment of whole body glucose homeostasis in mice fed HFD.

## Materials and Methods

### Animals

All procedures were approved by the Animal Care and Use Committee at the University of Virginia. Mice were provided food and water *ad libitum* and housed in 12/12 light/dark cycle at 68–72°F. Whole body CypD KO mice were obtained from Prof. Jeffrey Molkentin [19]. Skeletal muscle-specific KO (MKO) was generated by crossing muscle creatine kinase (MCK)-Cre mice (The Jackson Laboratory) with floxed-CypD (CypD^f/f^) mice (The Jackson Laboratory); Liver specific KO (LKO) was generated by crossing albumin-Cre mice (The Jackson Laboratory) with CypD^f/f^ mice. The high-fat diet (45% kcal as fat) was purchased from Research Diets (D12451) and fed to mice at 8 weeks of age for up to 14 weeks.

### Calcium Retention Capacity Assay

**Fig 1:**
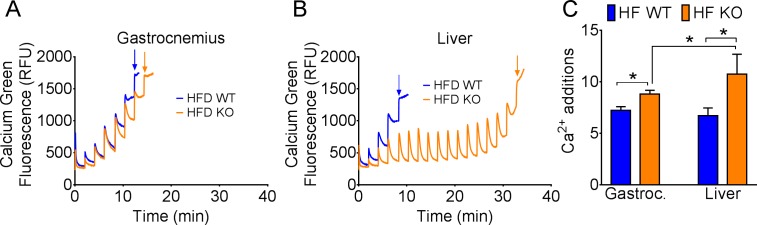
CypD is more important for calcium overload induced mitochondrial permeability transition in the liver compared with skeletal muscle. Representative calcium retention capacity assay of a single experiment on isolated mitochondria from skeletal muscle (A) and liver (B) of WT and CypD KO mice fed HFD for 10 weeks. (C) The average number of calcium additions required before irreversible mPTP opening was observed as indicated by fluorescence plateau and quantified for skeletal muscle and liver mitochondria. For gastrocnemius WT = 9, KO = 5; for liver WT = 7 and KO = 4. *p < 0.05.

Calcium retention capacity assay was performed in mitochondria isolated from liver and gastrocnemius muscle of CypD KO mice or wild type littermates (WT) fed HFD for 10 weeks as previously described [23, 28–29]. Tissues were homogenized with 5 ml of sterile isolation buffer (150 mM sucrose, 75 mM KCl 50 mM Tris-base, 1 mM KH_2_PO_4_, 5mM MgCl_2_, 1 mM EGTA and 0.2% BSA, pH 7.4). Nagarse (5 mg/ml) was added before homogenization and incubated on ice for exactly 1 min. Samples were homogenized on ice at the lowest speed for 3 x 15 sec with a 10 sec break in between. Fifteen ml of isolation buffer was added, and samples centrifuged at 700 x g for 10 min at 4°C. The supernatant was then centrifuged at 10,000 x g for 10 min at 4°C. The pellet was resuspended in 15 ml of suspension buffer (250 mM sucrose, 10 mM Tris-base, 0.1 mM EGTA, pH 7.4) and centrifuged at 8,000 x g for 10 min at 4°C. The mitochondrial pellet was resuspended in 50 μl of suspension buffer and kept on ice. A protein assay was performed to determine protein concentration. In a 96 well plate isolated mitochondria (15 μg) were suspended into a total volume of 100 μl using mitochondrial challenge buffer (250 mM sucrose, 10 mM MOPS, 0.05 mM EGTA, 10 mM Tris, pH 7.4) with the addition of 50 mM of Na-succinate (Sigma S2378) and 10 μM rotenone (Sigma R8875). One μl of Calcium Green 5N (100 μM; Invitrogen C3737) was added, and incubated at room temperature for 5 min. Measurements were then started with calcium pulse (83 nmol/mg protein) every 2 min until a plateau of fluorescence was observed. The fluorescence was measured using an excitation/emission wavelength of 506/532 nm, respectively, in a FLUOstar omega plate reader (BMG Labtech). We performed quantification of calcium retention capacity by counting the number of calcium additions required to induce opening of the mPTP, observed by a sharp increase in Calcium Green fluorescence following calcium addition (Shown on the representative Fig 1A and 1B by arrows).

### GTT and PTT

Glucose tolerance test was performed following 6 hours fasting as described previously [23] with i.p. injection of 2 g/kg glucose at normal chow fed mice and 1.5 g/kg glucose for HFD fed mice. Blood glucose was measured from the tail using a blood glucometer (Bayer) prior to glucose injection and at 15, 30, 60 and 90 min after. Pyruvate tolerance test was performed under the same conditions, but with i.p. injection of 2 g/kg pyruvate, and blood glucose was measured at the same time points.

### Insulin Stimulated Akt Phosphorylation in Skeletal Muscle

At the time of tissue harvest mice were anesthetized using 3% isoflurane and maintained under deep anesthesia with 2% isoflurane. The plantaris muscle was carefully dissected from the left leg and homogenized in western blot sample buffer. Insulin (5 U/kg) was injected i.p., and 10 minutes later the contralateral plantaris muscle was dissected and immediately homogenized in sample buffer. Total and phosphorylated Akt (Ser473) were measured by western blot as described below.

### Immunoblotting analysis

Tissues homogenates were prepared as previously described [30]. Thirty μg of total protein was subject to SDS-PAGE and transferred onto PVDF membrane. Membranes were probed with the following primary antibodies: mouse anti-CypD (AbCam, ab110324), rabbit anti-ANT (AbCam, ab180715), rabbit anti-VDAC (CST, #4866), mouse anti-ATP Synthase (Abcam, ab5432), mouse anti-phospho-Akt S473 (CST, #4051), rabbit anti-Akt (CST, #4691), mouse α-Tubulin (Abcam, ab11304). Secondary antibodies were goat anti-mouse IR680 (LICOR) and goat anti-rabbit IR800 (LICOR). Membranes were scanned using the Odyssey infrared imaging system (LICOR).

### Oil Red O Staining of Liver Sections

Fresh liver tissue was frozen in optimal cutting temperature compound (OCT) chilled in freezing isopentane. Five μm sections were cut using a cryostat and fixed in 10% buffered neutral formalin. Slides were rinsed in distilled water followed by a 2 min-incubation in absolute propylene glycol and a 16 hr-incubation in Oil Red O solution. Slides were then incubated in 85% propylene glycol for 1 min, rinsed in distilled water and stained in Mayer’s hematoxylin for 15–60 sec. Glass cover slips were mounted in aqueous mounting medium, and 10 images per slide were acquired using a bright field microscope. Oil Red O staining was quantified using ImageJ to assess lipid droplet size as previously described [31].

### Statistical Analysis

Data are expressed as mean ± standard error of the mean. Repeated measures ANOVA was used to determine statistically significant differences between genotype during GTT and PTT. Two-way ANOVA or two-tailed Student’s t-test were used as appropriate to determine statistically significant differences between genotype and diet. *P* < 0.05 was considered statistically significant.

## Results

### CypD is required for calcium overload-induced mPTP opening in liver mitochondria, but not in skeletal muscle mitochondria

In order to ascertain tissue specificity of mPTP and the importance of CypD, we performed calcium retention capacity (CRC) assays in mitochondria isolated from liver or gastrocnemius muscle of WT and CypD KO mice following 10 wk of HFD. In skeletal muscle mitochondria, loss of CypD had a very minimal impact on CRC requiring only 1 to 2 more additions of Ca^2+^ (Fig 1A and 1C). Loss of CypD in the liver mitochondria had a profound impact on CRC such that up to 12 rounds of Ca^2+^ addition (83 nM per mg mitochondrial protein) were required to trigger Ca^2+^ overload-induced mPTP opening compared to WT mitochondria (Fig 1B and 1C). These data suggest that CypD has a greater role in liver mPTP activity than skeletal muscle.

### Loss of CypD in skeletal muscle or liver does not alter expression of other mPTP regulatory components

**Fig 2:**
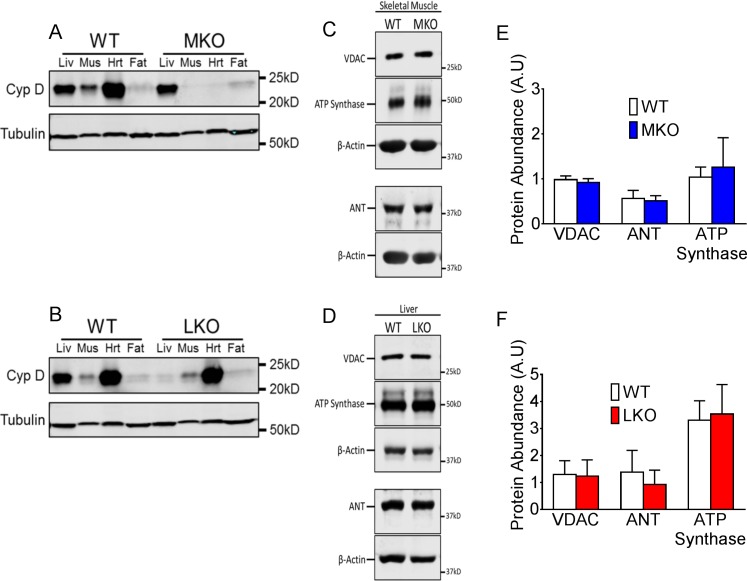
Deletion of CypD does not alter other mPTP protein abundance. (A) Western blot for CypD protein in liver, plantaris muscle, heart and epididymal fat tissue. MKO mice have muscle-specific deletion of CypD in skeletal muscle and heart (A), while LKO mice have liver-specific deletion (B). Littermate floxed mice are used as WT showing normal CypD expression. Representative western blot and quantification, respectively, in skeletal muscle (C and E) and liver (D and F) for VDAC, ATP Synthase and ANT. Quantification is normalized to the loading control, β-actin. *p < 0.05; n = 4–6.

To determine the importance of mPTP in skeletal muscle versus liver in the context of whole body physiology, we generated muscle-specific (MKO) and liver-specific (LKO) CypD knockout mice by crossing muscle creatine kinase (MCK)-Cre mice or liver albumin-Cre mice with CypD^f/f^ mice, respectively. Muscle-specific and liver-specific CypD deletion was confirmed by immunoblot in liver, skeletal muscle, heart and fat of MKO mice (Fig 2A) and LKO mice (Fig 2B). Deletion of CypD in either the skeletal muscle or liver had no impact on protein levels of other mPTP constituents including VDAC, ATP Synthase and ANT in the respective tissues (Fig 2C–2F).

### Loss of CypD in skeletal muscle does not alter susceptibility to HFD

**Fig 3:**
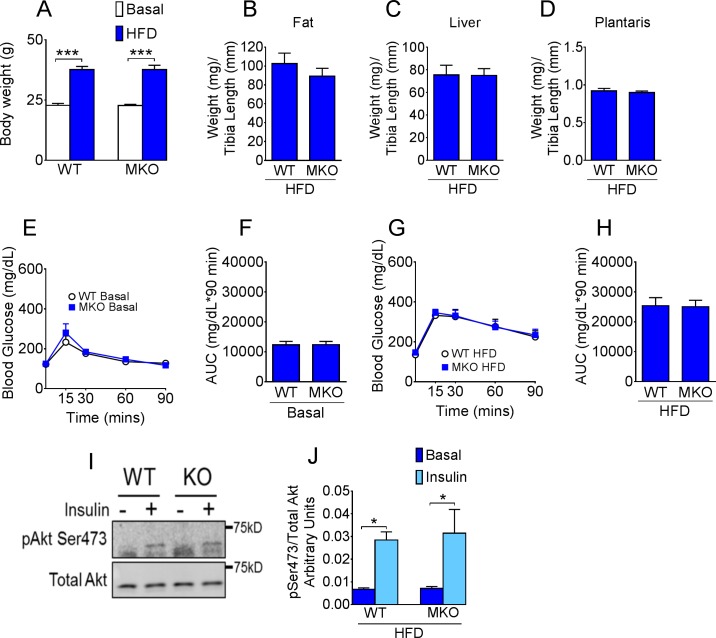
CypD deletion in skeletal muscle does not affect whole body glucose homeostasis. (A) Body weight at basal levels and following 11 wk of HFD; (B) Epididymal fat; (C) liver and (D) plantaris muscle tissue weight normalized to tibia length in HFD fed mice; (E) Blood glucose following i.p. injection of glucose in normal chow fed mice and (F) area under the glucose curve; (G) Blood glucose following i.p. injection of glucose in HFD fed mice and (H) area under the glucose curve; (I) Representative western blot and (J) quantification of phosphorylated (S473) and total Akt in plantaris muscle of HFD fed WT and MKO mice before and 10 min after i.p. injection of insulin. ***p < 0.001 basal vs. HFD; *p < 0.05 basal vs. insulin; n = 10–12.

Deletion of the CypD gene in muscle had no impact on body weight or weight gain following 11 weeks of HFD (Fig 3A). Nor did it affect epididymal fat, liver and skeletal muscle tissue weights (Fig 3B–3D). Whole body glucose metabolism (assessed by GTT) were similar between WT and MKO mice under both normal chow conditions (Fig 3E and 3F) and following 11 weeks of 45% HFD (Fig 3G and 3H). To further determine insulin sensitivity in skeletal muscle, we measured Akt phosphorylation of Ser473 in plantaris muscle at the baseline and 10 minutes after i.p. injection of insulin in these mice. Insulin signaling was unaffected by loss of CypD in skeletal muscle as shown by similar levels of induction of Akt phosphorylation (Ser473) by insulin injection in MKO compared with WT mice (Fig 3I and 3J).

### Loss of CypD in the liver causes metabolic dysregulation and increased fat deposition in mice fed HFD

**Fig 4:**
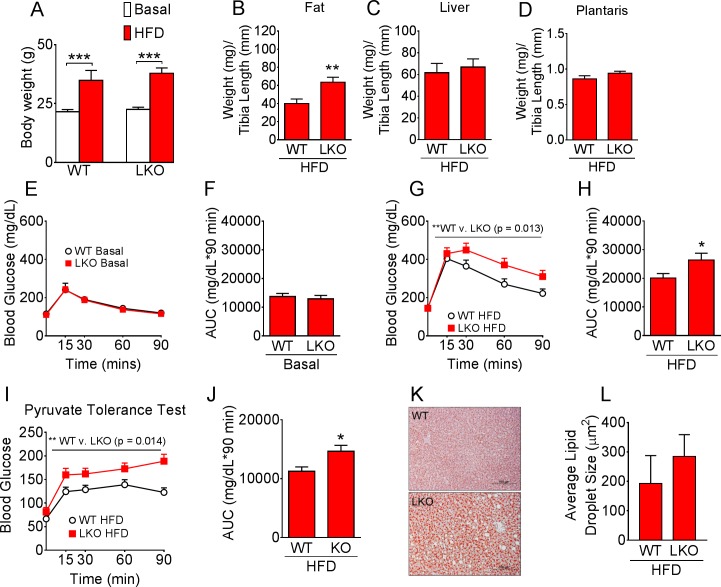
CypD is important for metabolic control in the liver under high-fat diet conditions. (A) Body weight at basal levels and following 11 wk of HFD; (B) Epididymal fat, (C) liver and (D) plantaris muscle tissue weight normalized to tibia length in HFD fed mice; (E) Blood glucose following i.p. injection of glucose and (F) area under the glucose curve under normal chow conditions; (G) Blood glucose following i.p. injection of glucose and (H) area under the glucose curve following 11 wk of HFD; (I) Blood glucose following i.p. injection of pyruvate and (J) area under the curve following 14 wk of HFD; (K) Representative Oil red O staining of liver sections from WT and LKO mice following 14 wk of HFD and (L) average lipid droplet size. ***p < 0.001 basal vs. HFD, * and **p < 0.05 WT vs. LKO; GTT and PTT n = 9–11; Oil Red O quantification n = 4.

LKO mice had similar body weight under normal chow conditions and following HFD compared with WT mice (Fig 4A). LKO mice, however, had significantly greater epididymal fat mass (Fig 4B), but similar liver weight (Fig 4C) and plantaris muscle weight (Fig 4D) following HFD. Although glucose tolerance was not different between WT and LKO mice at the basal level (Fig 4E and 4F), LKO mice displayed glucose intolerance with higher glucose during GTT compared with WT mice following 11 weeks of 45% HFD (Fig 4H and 4I). Importantly, the exacerbated metabolic dysfunction observed in LKO mice during GTT was of liver origin as evidenced by pyruvate tolerance test performed after 14 weeks of HFD (Fig 4I and 4J). The increase in blood glucose following pyruvate injection reflects hepatic gluconeogenesis, which appears to be poorly controlled in LKO mice. We did not observe significant differences in liver Oil Red O stained lipid droplet size, mostly due to small sample size and large variation (Fig 4K and 4L)

## Discussion

Our objective was to dissect the tissue specific role of CypD in the control of mPTP and metabolism in the liver and skeletal muscle. The vastly enhanced calcium retention capacity in isolated mitochondria from KO liver compared with WT liver after HFD suggests that CypD plays a critical role in the regulation of mPTP opening and hence mitochondrial function in the liver. The fact that liver-specific CypD knockout mice had exacerbated glucose intolerance suggests that normal mPTP in hepatocytes is critical for them to deal with lipid overload and maintain metabolic homeostasis under the condition of HFD. Surprisingly, calcium retention capacity was only marginally enhanced in KO skeletal muscle compared with WT skeletal muscle after HFD. Although CRC in WT mitochondria was similar between liver and skeletal muscle, we observed a considerable difference between liver and skeletal muscle mitochondria when the CypD gene was deleted, and the findings are suggestive of a tissue-specific role of CypD in mPTP regulation in response to calcium overload. Although our western blot data in WT mice suggests that CypD is much more abundant in liver compared with skeletal muscle, it is important to note that this analysis was performed on whole tissue lysates with equal protein loading and the majority of skeletal muscle protein consists of structural elements. Therefore differences in mitochondrial abundance between tissues may account for the difference in CypD abundance, and we cannot use these observations to explain those from the CRC assay, which was performed with equal amounts of isolated mitochondria. Importantly, these findings paved the way for us to use molecular genetic intervention to ascertain tissue-specific function of CypD in diet-induced obesity and metabolic syndrome in mice.

We generated mice with tissue-specific deletion of the CypD gene in the liver or muscle with the goal to determine CypD/mPTP contribution to metabolic homeostasis *in vivo* in a tissue specific manner. When the CypD gene was specifically deleted in muscle we observed no impact on whole body glucose tolerance, or on skeletal muscle insulin signaling assessed by Akt phosphorylation. These findings are perhaps not surprising considering the marginal impact of CypD gene deletion on isolated mitochondrial CRC from skeletal muscle. In fact, recently reported findings from *Shang* and colleagues (2016) showed that transient opening of the mPTP, causing ‘mitoflashes’, also displayed tissue-specific CypD dependency. They showed that frequency of mitoflashes was dramatically reduced in heart mitochondria lacking CypD, but was unaffected in skeletal muscle upon CypD deletion [32]. These findings support our current data that mPTP opening in skeletal muscle mitochondria can occur largely independently of CypD. It is important to note, however, that in the current study CypD MKO mice carry the MCK-Cre, which has leaking expression in the heart, and therefore the CypD protein is also deleted in cardiac tissue. Whether there are alterations in mitochondrial morphology and function in the heart or effects on cardiovascular health has not been assessed in this study.

In contrast to our findings in skeletal muscle, liver-specific deletion of the CypD gene resulted in exacerbated glucose intolerance and exaggerated hepatic glucose production under the condition of HFD. The impaired whole body glucose metabolism, particularly the elevated blood glucose in LKO mice may promote storage in fat tissue and account for the increased epididymal fat deposition that we observed in these mice. The increased fat deposition may in turn contribute to the impairment in whole body glucose tolerance in LKO mice. Furthermore, although the increase in blood glucose during the PTT in LKO mice is suggestive of a liver-specific phenotype, we cannot rule out a contribution of skeletal muscle to the metabolic phenotype since we did not assess skeletal muscle insulin sensitivity in these mice. Interestingly, a recent study reported that pyruvate administration significantly increased the frequency of transient mPTP opening in cardiomyocytes, which was 42% lower in CypD KO cells [30]. Although the impact of pyruvate administration on mPTP in liver is yet to be reported, the potential dampening of pyruvate-induced transient mPTP may be a link to account for the increased gluconeogenesis observed during the pyruvate tolerance test in LKO mice. Others have reported that loss of CypD in MEF cells resulted in multiple defects in TCA cycle intermediates, without changes in the abundance of the ETC complexes [23]. This is important since we did not observe significant changes in electron transport chain proteins in the LKO liver (S1 Fig). A metabolomics approach may perhaps be particularly informative for future mechanistic investigations. Alternatively, loss of CypD in the liver may have an impact on the overall maintenance of the mitochondrial population. Indeed, previous studies suggest that irreversible mPTP opening is required for targeted mitochondrial removal via autophagy (mitophagy) [16]. Therefore impairment of mPTP opening in LKO mice may lead to defects in mitophagy initiation and signaling resulting in accumulation of damaged or dysfunctional mitochondria in liver cells. This may become detrimental for metabolic health when challenged with chronic HFD causing elevated mitochondrial stress.

The data presented in this study shows that CypD is a regulator of mPTP in the liver and is critical for whole body metabolic health under HFD conditions. It points to the importance of normal sensitivity to stimuli for mPTP opening in the maintenance of metabolic homeostasis in the liver under the condition of lipid overload, such as diet-induced obesity. Our previous findings of improved glucose tolerance in whole body CypD KO mice following chronic HFD [23] may be a consequence of the CypD gene deletion in other tissues (e.g. brain or adipose) that may be able to compensate for impaired liver mitochondrial function and metabolic homeostasis. The current findings are of clinical importance considering other identified functions of CypD in ischemia reperfusion injury that are particularly relevant for cardiomyocyte and neuronal cells [33–35]. Future studies, such as in peripheral arterial disease that mainly affects skeletal muscle, will need to consider other potential therapeutic targets.

## Supporting Information

S1 Fig Expression of OXPHOS protein subunits in livers from CypD LKO and WT control mice fed HFD. (A) Representative Western blots of liver samples from HFD-fed CypD LKO and WT mice probed with the MitoProfile Total OXPHOS Rodent WB Antibody Cocktail to measure expression of ATP Synthase (Complex V, ATP5A subunit) and ETC complexes I (NDUFB8 subunit), II (SDHB subunit), III (UQCRC2 subunit) and IV (MTCO1 subunit). Expression of 14-3-3 was used as a loading control. M is 10ug of rat heart mitochondria as a control for OXPHOS protein subunits. (B) Quantification of liver OXPHOS subunit expression normalized to 14-3-3; results from three independent blots with n = 4 WT and 6 CypD LKO.
(PDF)

S1 Appendix Excel spreadsheet of raw data. (XLSX)
