# Supplementary material for: The Mitochondrial Permeability Transition Pore Regulator Cyclophilin D Exhibits Tissue-Specific Control of Metabolic Homeostasis
Source: PLoS One. 2016 Dec 22;11(12):e0167910. doi: 10.1371/journal.pone.0167910 (PMC5179060; doi:10.1371/journal.pone.0167910)
Supplement: S1 Fig — (A) Representative Western blots of liver samples from HFD-fed CypD LKO and WT mice probed with the MitoProfile Total OXPHOS Rodent WB Antibody Cocktail to measure expression of ATP Synthase (Complex V, ATP5A subunit) and ETC complexes I (NDUFB8 subunit), II (SDHB subunit), III (UQCRC2 subunit) and IV (MTCO1 subunit). Expression of 14-3-3 was used as a loading control. M is 10ug of rat heart mitochondria as a control for OXPHOS protein subunits. (B) Quantification of liver OXPHOS subunit expression normalized to 14-3-3; results from three independent blots with n = 4 WT and 6 CypD LKO. (PDF) [file pone.0167910.s001.pdf]

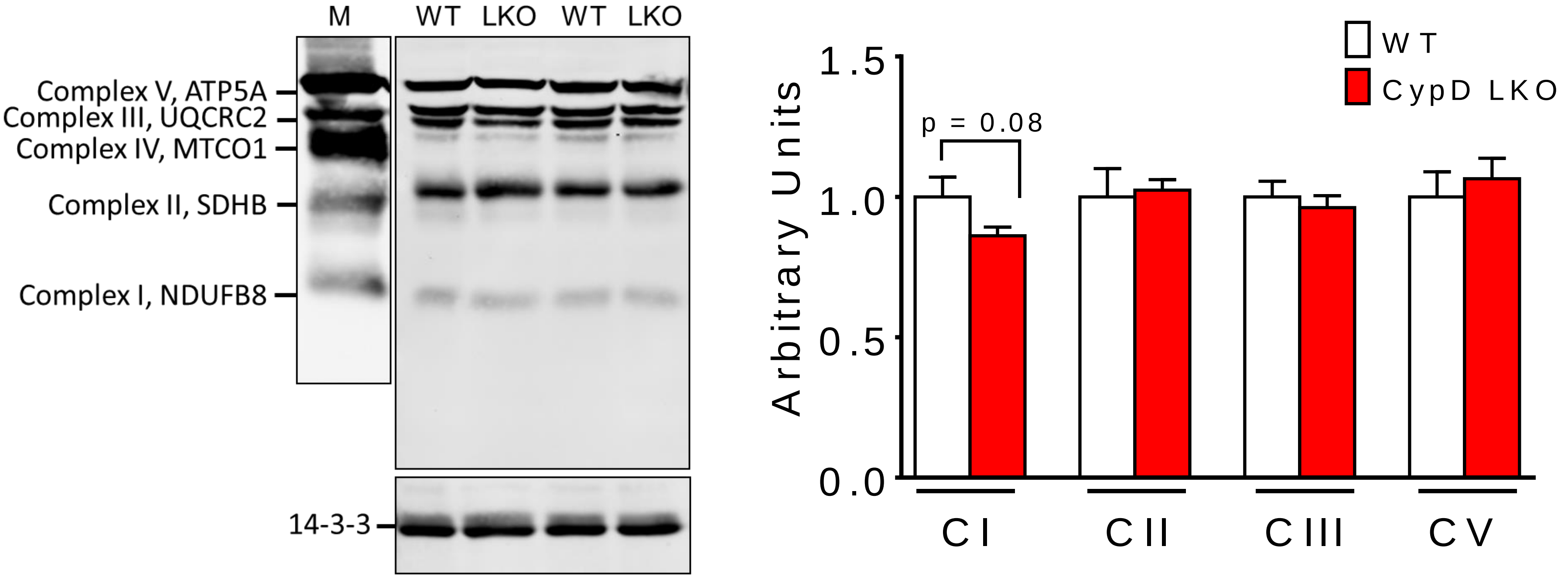

S1 Fig. Expression of OXPHOS protein subunits in livers from CypD LKO and WT control mice fed HFD. (A) Representative Western blots of liver samples from HFD-fed CypD LKO and WT mice probed with the MitoProfile Total OXPHOS Rodent WB Antibody Cocktail to measure expression of ATP Synthase (Complex V, ATP5A subunit) and ETC complexes I (NDUFB8 subunit), II (SDHB subunit), III (UQCRC2 subunit) and IV (MTCO1 subunit). Expression of 14-3-3 was used as a loading control. M is 10ug of rat heart mitochondria as a control for OXPHOS protein subunits. (B) Quantification of liver OXPHOS subunit expression normalized to 14-3-3; results from three independent blots with n = 4 WT and 6 CypD LKO.
